# Epipregnanolone as a Positive Modulator of GABA_A_ Receptor in Rat Cerebellar and Hippocampus Neurons

**DOI:** 10.3390/biom11060791

**Published:** 2021-05-24

**Authors:** Julia Bukanova, Elena Solntseva, Rodion Kondratenko, Eva Kudova

**Affiliations:** 1 Functional Synaptology Laboratory, Brain Research Department, Research Center of Neurology, Volokolamskoe Shosse 80, 125367 Moscow, Russia; julia@fast-mail.org (J.B.); kondrat_r@mail.ru (R.K.); 2 Institute of Organic Chemistry and Biochemistry of the Czech Academy of Sciences, Flemingovo Namesti 2, 160 00 Prague, Czech Republic

**Keywords:** GABA receptor, epipregnanolone, allopregnanolone, isopregnanolone, flumazenil patch clamp

## Abstract

Epipregnanolone (3β-hydroxy-5β-pregnan-20-one, Epi) is an endogenous steroid with important physiological effects and high affinity for GABA_A_ receptors. The effect of Epi on GABA-induced chloride current (*I*_GABA_) in native neurons has hardly been studied. In this work, we studied the influence of Epi on the *I*_GABA_ in the Purkinje cells of rat cerebellum and pyramidal neurons of rat hippocampus with the patch clamp technique. We showed that Epi is a positive modulator of the *I*_GABA_ with EC_50_ of 5.7 µM in Purkinje cells and 9.3 µM in hippocampal neurons. Epi-induced potentiation of the *I*_GABA_ was more potent at low vs. high GABA concentrations. Isopregnanolone (3β-hydroxy-5α-pregnan-20-one, Iso) counteracted Epi, reducing its potentiating effect by 2–2.3 times. Flumazenil, a nonsteroidal GABA_A_ receptor antagonist, does not affect the Epi-induced potentiation. Comparison of the potentiating effects of Epi and allopregnanolone (3α-hydroxy-5α-pregnan-20-one, ALLO) showed that ALLO is, at least, a four times more potent positive modulator than Epi. The combined application of ALLO and Epi showed that the effects of these two steroids are not additive. We conclude that Epi has a dual effect on the *I*_GABA_ increasing the current in the control solution and decreasing the stimulatory effect of ALLO.

## 1. Introduction

**Figure 1:**
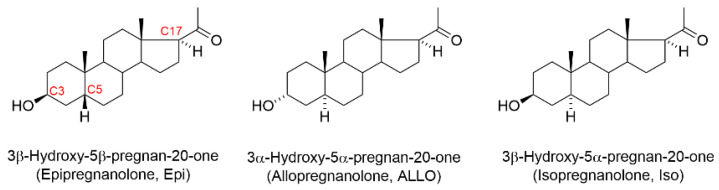
Structures of epipregnanolone (Epi), allopregnanolone (ALLO), and isopregnanolone (Iso). When the rings of a steroid are denoted as projections onto the plane of the paper, the α-substituent (hashed bond) is located below the plane, and the β-substituent (bold bond) above the plane of the paper. The important ring numbering positions are highlighted in red for epipregnanolone.

Steroids with pregnane skeleton were shown to be effective modulators of neuronal gamma-aminobutyric acid (GABA_A_) receptors [1,2]. The spatial arrangement of the hydroxyl group at C3 position of steroid skeleton (Figure 1) dramatically affects the biological properties of the steroids [3]. It has been shown that 3α-hydroxypregnanes (for example, allopregnanolone, ALLO,) potentiate the GABA-induced chloride current (*I*_GABA_) in nanomolar concentrations, while the 3β-hydroxy-pregnanes, i.e., epipregnanolone (Epi) and isopregnanolone (Iso) exhibit antagonistic properties and reduce the stimulating effect of 3α-hydroxypregnanes on *I*_GABA_ [4]. However, note that the literature describing the effect of 3β-hydroxypregnanes on *I*_GABA_ is controversial. The majority of studied 3β-hydroxy compounds were described to be effectively inert [5,6,7]. In contrast, some 3beta-hydroxy compounds, including Epi, have been found to induce mild positive modulatory effects. For example, Wang et al. [4] have found that *I*_GABA_ recorded from recombinant GABA_A_ receptors expressed in frog oocytes is potentiated by 30% in the presence of 10 µM Epi. Kokate et al. [8] showed that *I*_GABA_ in cultured hippocampal neurons was enhanced by 45% in the presence of 1 µM Epi. Strömberg et al. [9] investigated the effects of the Epi and its interaction with ALLO by recording spontaneous inhibitory postsynaptic currents (sIPSCs) in rat hypothalamic neurons. Both ALLO (100 nM) and Epi (3 µM) produced a marked prolongation of sIPSCs decay. Co-application of ALLO and Epi induced a 20–25% reduction of decay. The authors concluded that Epi can act both as a positive and negative GABA_A_-receptor modulator. However, this conclusion is not confirmed by other authors, who did not find signs of positive modulation of the *I*_GABA_ by Epi on the cells of the dorsal ganglia [10], recombinant GABA_A_ receptors [7], and pituitary melanotrope cells [6]. The ability of Epi to positively modulate *I*_GABA_ in native neurons has hardly been studied. The aim of this work was to study the effect of Epi on the *I*_GABA_ on neurons isolated from rat cerebellum and hippocampus.

Epi is the stereoisomer of ALLO, as it differs by its orientation of C-3 hydroxy group as compared to ALLO. ALLO has been implicated in a range of neurological and psychiatric diseases [11]. Epi, on the other hand, and for whatever reason, does not attract scientists to conduct further research. Nonetheless, the published studies clearly show a great therapeutic potential of Epi that could reach beyond modulation of GABA_A_ receptors. For example, O’Dell et al. [12] showed significant reduction of alcohol self-administration following pre-treatment with Epi. The authors proposed that this effect could be related to Epi’s actions on either GABA_A_ or *N*-methyl-*D*-aspartate (NMDA) receptors. Other authors [10,13] described analgesic properties of Epi. In the opinion of these authors, Epi does not significantly affect GABA_A_ receptors and the analgesic properties of this steroid are explained by its inhibitory effect on voltage-dependent Cav3.2 T-type calcium channels. We believe that our extensive study on Epi´s modulation of GABA_A_ receptors will offer a novel perspective of the mechanisms of its physiological effects.

## 2. Materials and Methods

### 2.1. Cell Preparation

All procedures were performed in accordance with the institutional guidelines on the care and use of experimental animals set by the Russian Academy of Sciences. The cells were isolated from transverse cerebellar or hippocampal slices as described in detail elsewhere [14]. Briefly, the slices (200–500 µm) of the Wistar rat (11–14 days of age) hippocampus or cerebellum were incubated at room temperature for at least 2 h in a solution containing the following components (in mM): 124 NaCl, 3 KCl, 2 CaCl_2_, 2 MgSO_4_, 25 NaHCO_3_, 1.3 NaH_2_PO_4_, 10 d-glucose, pH 7.4. The saline was continuously stirred and bubbled with carbogen (95% O_2_ + 5% CO_2_). Single pyramidal neurons from CA3 of hippocampus or Purkinje cells from cerebellum were isolated by a vibrating fused glass pipette with a spherical tip. In each experiment we used two animals. We obtained 3–4 slices from an animal and one cell from one slice. Data points represent an average from 6 to 7 cells.

### 2.2. Current Recordings

GABA-activated currents (*I*_GABA_) in isolated neurons were induced by a step application of agonist for 1–2 s with 40 s intervals through a glass capillary, 0.1 mm in diameter, which could be rapidly displaced laterally. Transmembrane currents were recorded using a conventional patch clamp technique in the whole-cell configuration. Patch clamp electrodes had a tip resistance of ~2 MΩ. The solution in the recording pipette contained the following (in mM): 40 CsF, 100 CsCl, 0.5 CaCl_2_, 5 EGTA, 3 MgCl_2_, 4 NaATP, 5 HEPES, 4 ATP, pH 7.3. The composition of extracellular solution was as follows (in mM): 140 NaCl, 3 KCl, 3 CaCl_2_, 3 MgCl_2_, 10 d-glucose, 10 HEPES hemisodium, pH 7.4. The speed of perfusion was 0.6 mL/min. The currents were recorded using an EPC7 patch clamp amplifier (HEKA Electronik, Reutlingen, Germany). The holding potential was maintained at −70 mV. Transmembrane currents were filtered at 3 kHz, stored and analyzed with an IBM–PC computer (Avelon, Moscow, Russia), using homemade software.

### 2.3. Reagents

All reagents used for intracellular and extracellular solutions as well as flumazenil were purchased from Sigma-Aldrich (St. Louis, MO, USA). Steroids were purchased from the following vendors: epipregnanolone (Carbosynth Ltd., Compton, Berkshire, UK, CAS 128-21-2, catalogue number FE177267), allopregnanolone (Carbosynth Ltd., Compton, Berkshire, UK, CAS 516-54-1, catalogue number FA158661), isopregnanolone (Steraloids, Newport, RI, USA, CAS 516-55-2, catalogue number P3830-000).

### 2.4. Data Analysis

Statistical analysis was performed by Prism GraphPad software (San Diego, CA, USA). All comparisons were made with unpaired (Section 3.1, Section 3.2, Section 3.3 and Section 3.5) or paired (Section 3.4) Student’s *t*-test at a significance level of *p* = 0.05. In the result descriptions, the mean and standard error of the mean (SEM) are specified. The EC_50_ value for steroid potentiation of the *I*_GABA_ was determined using the equation: Y = Bottom + (E_max_ − Bottom)/[1 + (EC_50_/C)^n^], where Bottom and E_max_ are current amplitudes measured in a control solution and in the presence of a steroid, respectively, C is the concentration of steroid, EC_50_ is the half-maximal stimulating concentration, and n is the slope factor (Hill coefficient).

## 3. Results

### 3.1. Epipregnanolone Potentiates the I_GABA_ in Purkinje Cells from Cerebellum

**Figure 2:**
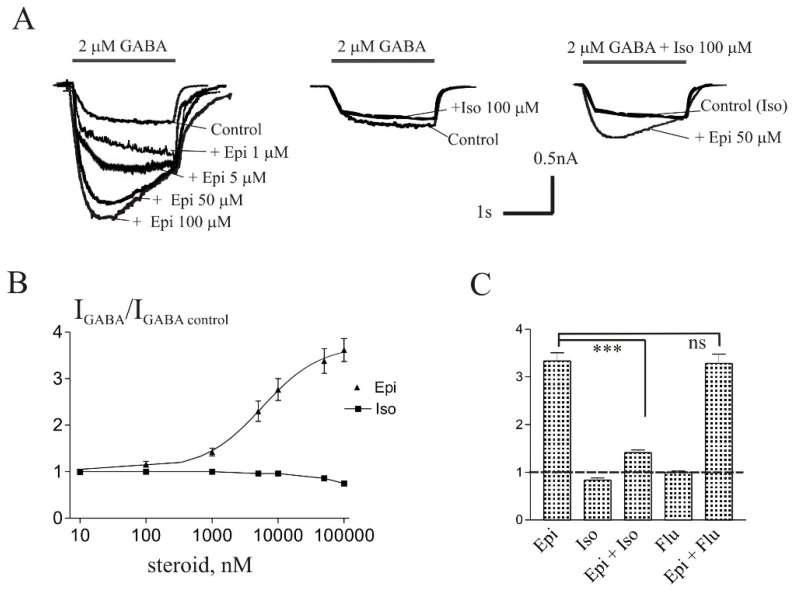
Iso antagonizes the potentiation of *I*_GABA_ induced by Epi in Purkinje cells. (**A**) Representative traces of the current induced by 2 µM gamma-aminobutyric acid (GABA) in control and during co-application with different concentrations of Epi (left), 100 µM Iso (middle) and 50 µM Epi together with 100 µM Iso (right). (**B**) Concentration dependence of Epi and Iso effect on the normalized peak amplitude of *I*_GABA_. (**C**) Mean ± standard error of the mean (SEM) of the normalized values of the peak amplitude of *I*_GABA_ in the presence of Epi (50 µM), Iso (100 µM), Epi + Iso, flumazenil (10 µM), Epi + flumazenil. The control value is indicated by a dashed line. Data points represent average from seven cells. *** Means *p* < 0.001, ns means not significant.

The brief application of GABA for 1–2 s on isolated Purkinje cells evoked a chloride current (*I*_GABA_) with an amplitude dependent on GABA concentration with an EC_50_ value of 6.8 ± 1.0 µM. The specific antagonist of GABA_A_ receptors bicuculline (3 µM) reversibly blocked the current. The average value of the reversal potential of *I*_GABA_ −9.7 ± 0.8 mV closely matched the chloride reversal potential calculated for the chloride concentrations used (−9.5 mV). Co-application of 2 µM GABA (EC_5_) with a different concentration of Epi caused the potentiation of the *I*_GABA_ (Figure 2). The potentiating effect of Epi developed slowly, particularly at low concentrations. The peak amplitude plotted in panels B was the highest point in the 2 s application.

The effect was reversible upon washout during 1–2 min. The threshold concentration of Epi was 1 µM, at which the peak amplitude of the current increased to 142 ± 8% of the control (*p* < 0.001, n = 7). A representative effect of Epi on *I*_GABA_ on one cell is shown in Figure 2A. An increase in the steroid concentration up to 100 µM caused a dose-dependent increase in the potentiation effect. The maximum effect (E_max_) was observed at 100 µM Epi and amounted to 372 ± 30% of the control, while the EC_50_ and Hill coefficient were 5.7 ± 1.9 µM and 0.98 ± 0.36, respectively. Figure 2B shows the concentration dependence of the Epi effect on the normalized *I*_GABA_ peak amplitude.

### 3.2. Isopregnanolone Antagonizes the Potentiating Effect of Epipregnanolone

Steroid Iso is a diastereoisomer of ALLO and was described to antagonize the potentiating effect of ALLO on the recombinant GABA receptors [4]. We studied whether Iso antagonizes the potentiating effect of Epi on the *I*_GABA_ in Purkinje cells. Co-application of 2 µM GABA with Iso at concentrations from 0.1 µM to 50 µM did not affect the *I*_GABA_. The only effective concentration of Iso was 100 µM at which the peak amplitude of the current reversibly decreased to 84 ± 4% of control (*p* < 0.05, n = 6). The representative traces of Iso effect on *I*_GABA_ on one cell is shown in Figure 2A. Figure 2B shows the concentration dependence of the Iso effect on the normalized *I*_GABA_ peak amplitude. Then we studied the effect of co-application of Epi and Iso. Low Iso concentrations (1–10 µM) did not change the Epi effect while higher Iso concentrations (50 and 100 µM) significantly (*p* < 0.001) weakened the effect of Epi. In particular, 50 µM Epi increased the *I*_GABA_ amplitude to 330 ± 17% of the control without Iso, to 167 ± 11% in the presence of 50 µM Iso, and to 141 ± 5% in the presence of 100 µM Iso. The representative traces of effect of Epi (50 µM) in the presence of Iso (100 µM) on *I*_GABA_ of one cell are shown in Figure 2A. Figure 2C shows the average normalized values of the peak amplitude of *I*_GABA_ in the presence of 50 µM Epi alone and in the presence of 100 µM Iso. The supposed mechanism of the weakening of the potentiating effect of Epi in the presence of Iso is a competitive inhibition for Epi ± Iso. In order to understand whether the benzodiazepine site of the GABA receptor is involved in the observed interaction of Epi and Iso, we used flumazenil, a known antagonist of the benzodiazepine site [15]. Our experiments demonstrated that 10 µM flumazenil does not affect the Epi-induced potentiation (Figure 2C).

### 3.3. The Effects of Epipregnanolone and Allopregnanolone on the I_GABA_ Are Not Additive

**Figure 3:**
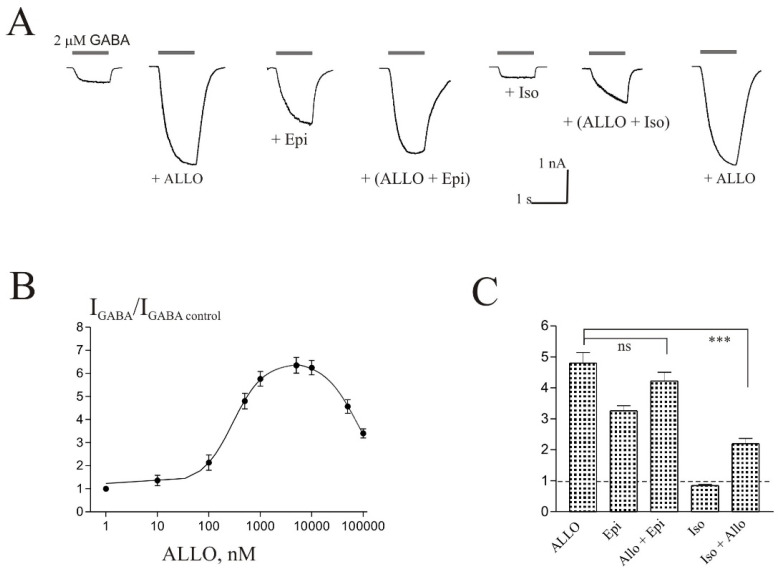
The effects of Epi and ALLO are not additive. (**A**) Representative traces of the current induced by 2 µM GABA in control and during co-application with 0.5 µM ALLO, 50 µM Epi, ALLO + Epi, 100 µM Iso, and ALLO + Iso. (**B**) Concentration dependence of ALLO effect on the normalized peak amplitude of *I*_GABA_. (**C**) Mean ± SEM of the normalized values of the peak amplitude of *I*_GABA_ in the presence of ALLO (0.5 µM), Epi (50 µM), ALLO + Epi, Iso (100 µM), Iso + ALLO. The control value is indicated by a dashed line. Data points represent average from seven cells. *** Means *p* < 0.001, ns means not significant.

ALLO is known to be a strong positive modulator of *I*_GABA_ in different cells [4,9,11]. In this work, we have shown for the first time that ALLO induces a strong potentiation of the *I*_GABA_ in the Purkinje cells of cerebellum (Figure 3). The representative traces of effect of 0.5 µM ALLO on *I*_GABA_ of one cell are shown in Figure 3A. Figure 3B shows the concentration dependence of the ALLO effect on the normalized *I*_GABA_ peak amplitude leading to a bell-shaped concentration–response. In particular, ALLO gradually enhanced *I*_GABA_ in the concentration range from 10 to 5000 nM, but at higher concentrations (10–100 µM) the steroid began to reduce the current. The E_max_ (646 ± 37%), EC_50_ (0.29 ± 0.06 µM), and Hill coefficient (1.5 ± 0.3) values were determined from the fit to the rising phase of the curve. Comparison of the effects of ALLO and Epi, taken at a concentration of 5 µM, shows that ALLO is at least 4 times more effective as a positive modulator of *I*_GABA_ on Purkinje cells. In the presence of 5 µM ALLO, the amplitude of the *I*_GABA_ increased by 535 ± 47% and in the presence of 5 µM Epi the amplitude of the *I*_GABA_ increased by 122 ± 15% (*p* < 0.001, n = 7). In the presence of Iso (50 or 100 µM), the effect of ALLO significantly decreased. According to the literature, Epi is able to suppress the effect of ALLO on the *I*_GABA_ [4,9]. We studied *I*_GABA_ on Purkinje cells with co-application of Epi and ALLO at an ALLO concentration of 0.5 µM and various Epi concentrations (1, 10, and 50 µM). We found that, in these experiments, the summation of the ALLO and Epi effects is violated. The absence of additivity of effects is especially clearly seen at high concentrations of Epi (50 µM) (Figure 3). A representative effect of 50 µM Epi in the presence of 0.5 µM ALLO on *I*_GABA_ of one cell is shown in Figure 3A. Figure 3C shows the average normalized values of the peak amplitude of *I*_GABA_ in the presence of 0.5 µM ALLO (482 ± 32% of control), 50 µM Epi (333 ± 17% of control), ALLO and Epi together (422 ± 23% of control), 100 µM Iso (84 ± 4% of control), and ALLO together with Iso (288 ± 27% of control).

### 3.4. Epipregnanolone-Induced Potentiation of the I_GABA_ Is More Efficacious at Low vs. High GABA Concentrations

**Figure 4:**
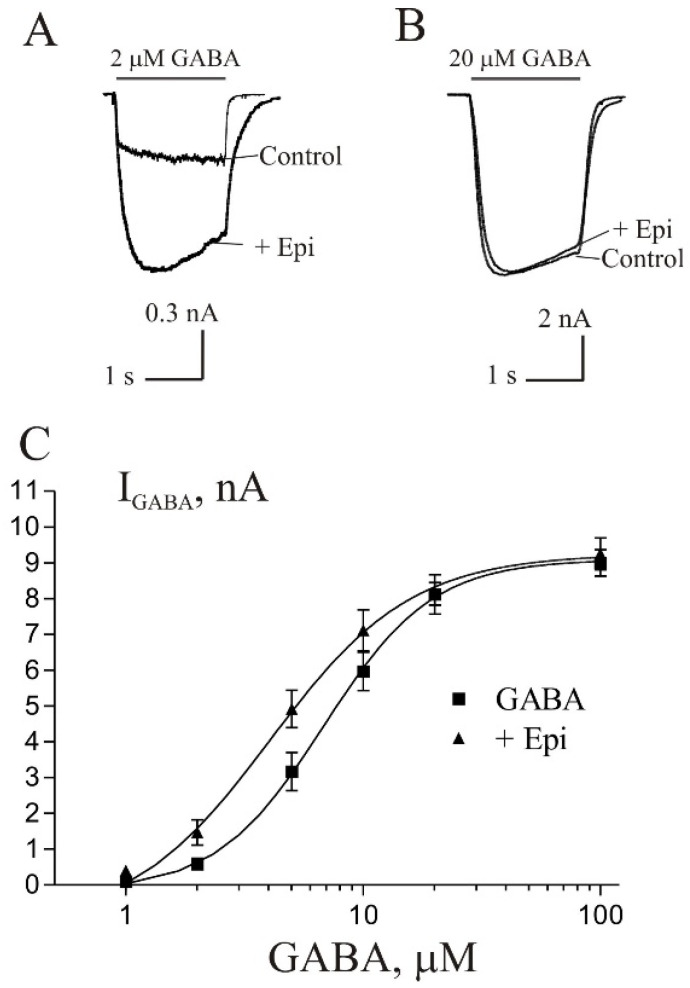
The degree of Epi effect depends on GABA concentration. (**A**,**B**) Representative traces of current induced by 2 µM GABA (**A**) and 20 µM GABA (**B**) in control and during co-application with 10 µM Epi. (**C**) Concentration–response curves for GABA obtained in control (squares) and in the presence of 10 µM Epi (triangles). Data points represent an average from six cells.

The influence of agonist concentration on the extent of Epi-induced potentiation was determined by measuring the potentiation by 10 µM Epi of currents evoked by increasing GABA concentration from 2 to 100 µM (Figure 4). The potentiation was GABA concentration-dependent, being larger at lower concentrations of GABA. Statistical analysis was performed using the paired Student’s *t*-test. The current induced by 2 µM GABA, was enhanced by Epi to 253 ± 26% of control (*p* < 0.05, n = 6) and current induced by 5 µM GABA was enhanced to 165 ± 19% of control (*p* < 0.05, n = 6). The potentiating effect was not observed at higher GABA concentrations (10, 20 and 100 M). The comparison of concentration–response curve for GABA in control and during co-application with 10 µM Epi shows that the steroid did not change the maximal GABA current but shifted dose–response curve to the left. The EC_50_ value was changed from 6.8 ± 1.0 µM in control condition to 4.1 ± 1.4 µM in the presence of 10 µM Epi (*p* < 0.001, n = 6). The difference in the values of the Hill coefficient is not statistically significant: 1.9 ± 0.5 in control and 1.45 ± 0.4 in the presence of 10 µM Epi.

### 3.5. Epipregnanolone Potentiates the I_GABA_ in Pyramidal Neurons from Hippocampus

**Figure 5:**
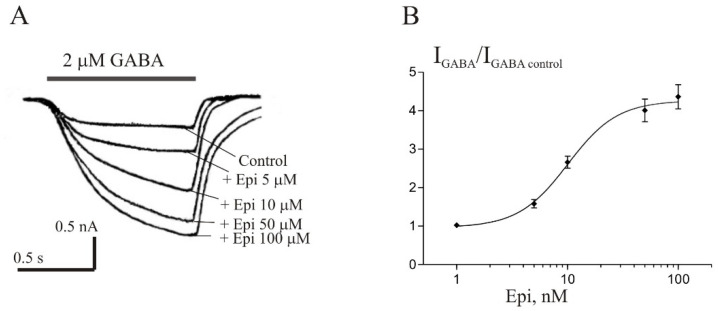
Epi potentiates the *I*_GABA_ in pyramidal neurons of rat hippocampus. (**A**) Representative traces of the current induced by 2 µM GABA in control and during co-application with different concentrations of Epi. (**B**) Concentration dependence of Epi effect on the normalized peak amplitude of *I*_GABA_. Data points represent average from six cells.

Strömberg et al. [9] found that the effects of Epi on GABA-induced Cl^−^ uptake were different in the rat cerebellum and hippocampus. However, in our experiments, no significant difference was found in the effects of Epi on *I*_GABA_ in neurons of the rat cerebellum and hippocampus. Isolated pyramidal neurons from CA3 area of hippocampus with high sensitivity to GABA were chosen for the experiments. The brief application of GABA for 1 s evoked the *I*_GABA_ in these neurons, whose amplitude was dependent on GABA concentration, with an EC_50_ value of 8.1 ± 2.8 µM. Co-application of 2 µM GABA with different concentration of Epi caused the potentiation of the *I*_GABA_ (Figure 5). The effect was reversible upon washout during 1–2 min. The threshold concentration of Epi was 5 μM, at which the peak amplitude of the current increased to 156 ± 11% of control (*p* < 0.01, n = 6). An increase in the steroid concentration up to 100 μM caused a dose-dependent increase in the potentiation effect. The maximum effect was observed at 100 µM Epi and amounted to 420 + 38% of the control, while the EC_50_ and Hill coefficient were 9.3 + 2.9 µM and 1.8 + 0.6, respectively.

## 4. Discussion

In the present work, we showed that Epi is a positive modulator of *I*_GABA_ in the Purkinje cells of the rat cerebellum (E_max_ = 372% and EC_50_ = 5.7 µM) and pyramidal neurons of the rat hippocampus (E_max_ = 420% and EC_50_ = 9.3 µM). The potentiating effect of Epi developed slowly, particularly at low concentrations. Li et al. [16] studied the reasons for the slow development of *I*_GABA_ potentiation in the presence of a steroid and concluded that the slow actions are not likely to result from slow kinetics of interaction with the GABA_A_ receptor, but rather reflect the slow equilibrium of steroid in a membrane compartment that is in equilibrium with the receptor. We further showed that Epi caused an increase in the affinity of the GABA_A_ receptors for the agonist in Purkinje cells. In the presence of Epi, the concentration dependence curve for GABA shifted to the left along the horizontal axis with a significant decrease in the EC_50_ value. Relative to ALLO, Epi was a significantly weaker positive modulator of *I*_GABA_. The EC_50_ value for ALLO was almost 20 times lower than for Epi (0.29 µM vs. 5.7 µM). The shape of the dose-response curves for Epi and ALLO was also different. In the concentration range 0.01–100 µM, this curve was smooth for Epi and bell-shaped for ALLO. At the same time, Iso weakened the potentiation caused by Epi and ALO in the same way while the benzodiazepine site antagonist flumazenil was inert.

Literature data on the ability of Epi to positively modulate the GABA_A_ receptors is contradictory. Using [^3^H]flunitrazepam binding assay, it was found that Epi can act as a partial agonist on a common neurosteroid modulatory site at the GABA_A_ receptor complex in avian central nervous system [17]. Electrophysiological experiments on recombinant GABA_A_ receptors expressed in Xenopus oocytes showed that *I*_GABA_ amplitude increased by 30% in the presence of 10 µM Epi [4]. The authors tested five 3β-hydroxysteroids and Epi was the only drug with the ability to potentiate the *I*_GABA_. Strömberg et al. [9] investigated the effects of Epi and ALLO by recordings spontaneous inhibitory postsynaptic currents (sIPSCs) in rat hypothalamic neurons. Epi (3 µM) and ALLO (0.1 µM) was found to act in a similar way, i.e., without changing the peak amplitude of sIPSCs, they slowed down their decay, so the decay time constant (τ_decay_) increased significantly. However, other authors did not find positive modulation of the *I*_GABA_ by Epi on the cells of the dorsal ganglia [10], recombinant GABA_A_ receptors [7], and in pituitary melanotrope cells [6]. Therefore, our work confirms Epi’s ability to potentiate *I*_GABA_ in native neurons.

It has been shown on different model systems that Epi suppress the potentiating effect of ALLO on the *I*_GABA_ [4,9,18]. Both competitive [18] and non-competitive [4] mechanisms of interaction of these steroids are discussed. Wang et al. [4] studied interaction between Epi and ALLO in recombinant GABA_A_ receptor and found that 10 µM Epi reduced effect of high (more than 3 µM) but not low concentrations of ALLO, which suggested noncompetitive mechanism. In our experiments, we observed the lack of additivity between Epi and ALLO at relatively low concentrations of ALLO (0.5 μM). It allows to suggest that the effects of steroids obtained on recombinant GABA_A_ receptor and on native GABA_A_ receptor in Purkinje cells may have different mechanisms and that our results, in our opinion, can be explained by the competition between Epi and ALLO for the same site. It is also important to note another difference between our data and the results of Wang et al. [4]. In our experiments, the concentration dependence of the ALLO effect on the *I*_GABA_ was bell-shaped, while this curve was smooth on recombinant GABA receptors.

GABA_A_ receptors are the major inhibitory receptors in the central nervous system, formed by combination of α1–6, β1–3, γ1–3, ρ1–3, ε, π, δ or θ subunits with the predominant receptor being α1β2γ2 with a subunit stoichiometry of 2:2:1 [19]. Despite the fact, that GABA_A_ receptors are the primary molecular targets of neurosteroid action, the structural details of neurosteroid binding to these proteins remain poorly understood. Multiple functional neurosteroid binding sites are believed to exist on GABA_A_ receptor [20]. Three neurosteroid-binding sites in the α1β3 GABA_A_ receptor are identified to contribute to neurosteroid allosteric modulation [1,3]. Sugasawa et al. [1] found that potentiating neurosteroid ALLO, but not its inhibitory 3β-epimer Epi binds to the canonical β3(+)–α1(−) inter-subunit site that mediates receptor activation by neurosteroids. In contrast, both ALLO and Epi bind to intrasubunit sites in the β3 subunit, promoting receptor desensitization. Another intrasubunit site in the α1 subunit promotes effects that vary between neurosteroids. The authors believe that differential occupancy and efficacy at three discrete neurosteroid-binding sites determine whether a neurosteroid has potentiating, inhibitory, or competitive antagonist activity on GABA_A_ receptors. It can be thought that the same neurosteroid is capable of interacting with at least two sites on GABA_A_ receptor. The bell-shaped dependence of the ALLO effect on concentration observed in our experiments indicates that the drug interacts with two sites, potentiating and desensitizing. At the same time, smooth dependence of the Epi effect on concentration suggests its interaction with one potentiating site. We believe that ALLO and Epi interact with the same potentiating site. This assumption is confirmed in the work of Shin et al. [21]. The authors provide functional evidence that 5α- and 5β-reduced steroids interact with the same sites on the GABA_A_ receptor. During co-application of two positive modulators acting at the same sites, the nature of GABA_A_ receptors modulation depends on the efficacies and concentrations of each compound. In our experiments with co-application of ALLO and Epi, the concentration ratio was strongly in favor of Epi. It seems that the latter displaces ALLO from its binding sites and this affect the response amplitude.

## Data Availability

The data presented in this study are available on reasonable request from the corresponding author synaptology@mail.ru (E.S.).
